# Brain tissue oxygen dynamics while mimicking the functional deficiency of interneurons

**DOI:** 10.3389/fncel.2022.983298

**Published:** 2022-10-20

**Authors:** Daniil P. Aksenov, Evan D. Doubovikov, Natalya A. Serdyukova, David A. Gascoigne, Robert A. Linsenmeier, Alexander Drobyshevsky

**Affiliations:** ^1^Department of Radiology, NorthShore University HealthSystem, Evanston, IL, United States; ^2^Department of Anesthesiology, NorthShore University HealthSystem, Evanston, IL, United States; ^3^Pritzker School of Medicine, University of Chicago, Chicago, IL, United States; ^4^Department of Biomedical Engineering, Northwestern University, Evanston, IL, United States; ^5^Department of Pediatrics, NorthShore University HealthSystem, Evanston, IL, United States

**Keywords:** GABA, neurovascular unit, neuronal synchronization, cerebral cortex, rabbit, epilepsy, hypersynchronization

## Abstract

The dynamic interaction between excitatory and inhibitory activity in the brain is known as excitatory-inhibitory balance (EIB). A significant shift in EIB toward excitation has been observed in numerous pathological states and diseases, such as autism or epilepsy, where interneurons may be dysfunctional. The consequences of this on neurovascular interactions remains to be elucidated. Specifically, it is not known if there is an elevated metabolic consumption of oxygen due to increased excitatory activity. To investigate this, we administered microinjections of picrotoxin, a gamma aminobutyric acid (GABA) antagonist, to the rabbit cortex in the awake state to mimic the functional deficiency of GABAergic interneurons. This caused an observable shift in EIB toward excitation without the induction of seizures. We used chronically implanted electrodes to measure both neuronal activity and brain tissue oxygen concentrations (PO_2_) simultaneously and in the same location. Using a high-frequency recording rate for PO_2_, we were able to detect two important phenomena, (1) the shift in EIB led to a change in the power spectra of PO_2_ fluctuations, such that higher frequencies (8–15 cycles per minute) were suppressed and (2) there were brief periods (dips with a duration of less than 100 ms associated with neuronal bursts) when PO_2_ dropped below 10 mmHg, which we defined as the threshold for hypoxia. The dips were followed by an overshoot, which indicates either a rapid vascular response or decrease in oxygen consumption. Our results point to the essential role of interneurons in brain tissue oxygen regulation in the resting state.

## Introduction

A shift in excitatory-inhibitory balance (EIB) in the brain toward excitation is a very common clinical and pathophysiological phenomenon, which can present in adulthood or as a result of disturbed development, due to the functional deficiency of inhibitory interneurons (Gascoigne et al., 2021). This phenomenon manifests as neuronal hypersynchronization during the resting state, a process which affects normal neuronal and neurovascular interactions (Staley, 2015; van Vliet and Marchi, 2022). Note, that the term “hypersynchronization” referred to in this study is related to the epileptiform bursts in neuronal activity and is different from other types of synchronization (e.g., to stimulation, during sleep, etc.) (Traub et al., 1993; Behrens et al., 2005; Schneider et al., 2019). The clinical characteristics of a shift in EIB, range from autism (Rubenstein and Merzenich, 2003) to epilepsy (Kaeser and Regehr, 2014), depending on its severity. Typically, this state has a very long duration (months and years) (Berg, 2011; Gascoigne et al., 2021).

The changes to neurovascular interactions that occur as a result of a shift in EIB toward excitation are not well known. From a clinical perspective, the most important question pertains to whether this process leads to localized hypoxia, due to the significantly heightened energy consumption from synchronized neuronal activity. Previous studies have found neurovascular decoupling during synchronized neuronal activity, yet have not been able to report actual hypoxia (Winkler et al., 2012; Prager et al., 2019; Ferlini et al., 2021). Moreover, some studies have even shown increased cerebral blood flow (CBF) and decreased blood oxygenation during seizures (Kreisman et al., 1991; Bahar et al., 2006; Zhao et al., 2009). It should be noted that oxygen levels in these studies decreased slowly (during seconds) and remained within normoxic criteria, whereas clinical hypoxia is typically considered to be below 10–15 mmHg in brain tissue PO_2_ assuming the normal PO_2_ is 20–40 mmHg (Ougorets and Caronna, 2008; Manole et al., 2014; Farrell et al., 2016). In line with this, an interesting study, which used 1 Hz frequency recordings, reported drops in brain tissue oxygen levels in response to absence seizures of greater than 10 s in duration (Farrell et al., 2018), yet the PO_2_ stayed above 15 mmHg during seizures.

In our study, we have utilized an innovative approach which combines simultaneous neuronal/local field potential and high-frequency (20 Hz) brain tissue oxygen recording from the same location. This allows any changes in neuronal activity to be simultaneously linked with brain tissue oxygen. Moreover, our ability to perform localized microinjections (Aksenov et al., 2014) allows us to effectively locally modulate EIB in awake animals, without affecting the state of the whole brain or afferent projections to the area of interest. Using this approach, we mimicked the functional deficiency of GABA-ergic interneurons in the cerebral cortex using the GABA-antagonist, picrotoxin, to elicit the shift in EIB without generating seizures.

Our results indicate that the shift in EIB causes brief but repeated periods of hypoxia in brain tissue. The presence of such hypoxic events suggests similar processes in patients who experience this type of shift in EIB due to functional deficiency of interneurons.

## Methods

### Subjects and approach justification

We have used awake rabbit models extensively in previous studies for studying behavioral learning as well as for electrophysiology, brain tissue oxygen, and MR measurements (Aksenov et al., 2016, 2020). There is no discomfort or pain connected with the reported experiments. Thus, rabbits are ideal subjects for electrophysiological experiments.

We chose layer IV of the somatosensory cortex for electrode implantation and drug injections because this brain region receives direct projections from the thalamus and represents one of the main components of the thalamocortical circuits (Ramirez et al., 2014; Zhang and Bruno, 2019). The somatosensory cortex has been extensively examined previously in rabbits (Swadlow et al., 1998). We have chosen the GABA-antagonist picrotoxin because the effects of this drug are well characterized and only involve GABA receptors.

### Animal preparation

Seven adult female Dutch-belted rabbits (9-months old, 2–3 kg) were used in accordance with the National Institutes of Health guidelines and protocols, and all procedures, were approved by the NorthShore University HealthSystem Research Institute Institutional Animal Care and Use Committee. Rabbits were housed in standard stainless steel cages with water and food *ad lib*. Animals were chronically implanted with electrodes, as reported previously (Aksenov et al., 2015). For this procedure, animals were anesthetized with a mixture of ketamine (60 mg/kg) and xylazine (10 mg/kg). The recording assembly consisted of a silica tube (Polymicro Technologies, Phoenix, AZ, USA) containing a bundle of four 25 μm diameter gold-silver alloy microwires with formvar insulation (California Fine Wire, Grover Beach, CA, USA, alloy 446), and electroplated with a thin layer of gold (Dalic alkaline plating solution; Sifco Chemical, Independence, OH) at the tip so that they could be used as traditional polarographic oxygen electrodes. These electrodes terminated at different levels within a distance of 150–200 μm. The microwires were connected to a small 6-pin connector that was embedded in dental acrylic. A 150 μm Ag/AgCl reference wire was placed between the skull and dura mater. A 200 μm silica injection cannula was attached to the microdrive. During surgery, lambda was positioned 1.5 mm below bregma and the stereotaxic coordinates were as follows: anterior-posterior was 2 mm dorsal to bregma, medial-lateral was 6 mm from midline, and dorsal-ventral was under visual control. After implantation the electrode assembly was cemented to the skull using dental acrylic and nylon support screws. After recovery from surgery each subject was habituated for at least 3–5 days prior to the experiments and all experiments were performed beginning at least 14 days after surgery. During recordings the rabbits were restrained by means of a cloth sleeve but their head was not fixed to the cradle. The rabbit’s docile temperament and tolerance for restraint are ideal for studies performed in the awake state (Aksenov et al., 2019). The electrodes were advanced to layer IV and their location was confirmed using anatomical MR imaging.

### Experimental design

Picrotoxin (Sigma Aldrich, 166 μM) was injected after 15 min of baseline recording. This concentration was chosen because it induces neuronal hypersynchronization without actual seizures. The total duration of an experiment was 50 min. We followed a previously described procedure of localized injections (Aksenov et al., 2005). For the control injections, the same protocol was followed as in picrotoxin injections, but a control vehicle (saline) was injected.

All injections (1 μl volume) were delivered through a silica tube/needle connected to a Hamilton syringe using transparent Tygon tubing. The pH level was adjusted for all injections and was 7.2–7.4. Single units were monitored to ensure that the impact of the potential mechanical displacement of tissue by the injected volume (volume effect) was minimized (Aksenov et al., 2014).

### Electrophysiological and PO_2_ recording

Our design allowed us to use the same electrode to record either single units or PO_2_. The multiple unit signals [local field potentials (LFP) were recorded simultaneously] from the microwires were fed through a miniature preamplifier to a multi-channel differential amplifier system (Neuralynx, Inc, USA). The recording and analysis of electrophysiological data has been described previously (Aksenov et al., 2014, 2015). Briefly, the signals were amplified, band-pass–filtered (300 Hz to 3 kHz for single units (SU) and 1–150 Hz for LFP), and digitized (32 kHz/channel) using a Neuralynx data acquisition system. Unit discrimination was performed offline using threshold detection followed by a cluster analysis of individual action potential wave shapes using Neuralynx analysis software. For PO_2_ recording the microwire was polarized to −0.7 V with respect to a reference electrode (located between the skull and dura), and the current was measured with a Keithley model 614 electrometer (Keithley Instruments, Cleveland, OH, USA). The initial designation of which wire, was used for either PO_2_ or neuronal activity recording, was random. The output voltage from the electrometer was low-pass filtered at 30 Hz, amplified, and digitized at 20 Hz. The chronically implanted PO_2_ electrodes were calibrated before implantation. Additionally, to show that our results do not depend on the sampling rate and filters in terms of PO_2_ power spectra, we acquired the data at 200 Hz with the low pass filter at 400 Hz in 2 animals.

Electrode locations were confirmed by MRI using a 9.4T imaging spectrometer (BioSpec 94/30USR, Bruker Biospin MRI GmbH) operating at 1H frequency of 400 MHz. The spectrometer was equipped with an actively-shielded gradient coil (BFG-240–150-S-7, Research Resonance, Inc., Billerica, MA, USA). A single-turn, 20mm-diameter circular RF surface coil was used for both transmission and reception. Anatomical images were acquired using a multislice gradient echo pulse sequence with a TR of 1.5 s, a TE of 10 ms, a 30 mm × 30 mm FOV, and a matrix size of 128 × 128, corresponding to an in-plane resolution of 234 μm × 234 μm.

### Data processing

Our initial data consisted of PO_2_ data sampled at 20 Hz, and LFP and MUA data sampled at 32 kHz. Then we resampled the data to match the sampling frequencies by taking the necessary increments between data points. For our MUA data, we first took the absolute value over the entire time series, then we used a non-overlapping moving average, selecting the window sizes to produce our desired resampled sampling frequency. For our initial analysis, where we compared the time series dynamics of PO_2_ against the time series of both MUA and LFP, we resampled our initial data to 20 Hz. For our second stage of analysis, we resampled all our data to 10 Hz. In total, our picrotoxin (PTX) and saline data each consist of seven experiments (one of each per animal). Note that for plotting the LFP spectrogram, we used a sampling rate of 200 Hz. All pre-processing of the data was done using scripts in Matlab, 2017b in the Windows 10 OS. All subsequent analysis (with the exception of the LFP spectrograms, described below) was performed using iPython notebooks (running Python 3) in the Linux OS.

### Power spectra analysis

To study how the frequency content of our data changed over time, we used rolling windows to create spectrograms of our PO_2_ and LFP data. For PO_2_ at a sampling rate of 10 Hz, we created spectrograms using a time window size of 10 min (6,000 time points), with an overlapping window size of 5,999 time points. For each time window, we scaled the power spectra by dividing by the maximum power value across all frequencies, e.g., for the frequency range with the highest power, the scaled power was equal to 1. For LFP under a sampling rate of 200 Hz, we created spectrograms using a time window size of 10.24 s (2,048 time points), with an overlapping window of 1,024 time points. The units for the LFP spectrogram are in dB/Hz (power/frequency). Power spectra diagrams for PO_2_ were done in 10-min non-overlapping windows. For this paper, we display only the 0-to-10-min and 30-to-40-min time window for comparative purposes. All power spectra calculations are done using Welch’s method (Welch, 1967).

### Consumption and synchronization measures

Since it was stated that the normal PO_2_ in brain tissue is 20–40 mmHg and the critical level is 10–15 mmHg in patients (Ougorets and Caronna, 2008), we applied a proportion of 0.4 (i.e., 12/30) to our initial baseline level as the critical threshold. This enabled us to analyze the number of dips below “hypoxic” levels, which are called “consumption spikes” here.

Consumption spikes are characterized by sudden dips in PO_2_, beyond what can be considered standard fluctuations in oxygen. To identify these abnormalities, we created a new smoothed series, where we applied a moving average smoothing filter over our original PO_2_ time series, with a time window of 5 time points (0.5 s). We then created a residual series, which consisted of the differences between the original PO_2_ series and the smoothed series. We then normalized the residual series in accordance with a Gaussian distribution. Finally, for this study, we defined the time of increased O_2_ consumption by locating all the time points in the normalized residual series which exceeded the value of 5 (i.e., the PO_2_ series deviated from the smoothed series by 5 standard deviations). The high threshold of 5 standard deviations ensured a robust barrier to decrease the chance of recording a false positive case of consumption. Similarly, using our MUA series (resampled at 10 Hz, using averaged bins), we denoted the time points of MUA “synchronization,” by recording all of the time points which exceeded 5 standard deviations. Finally, we defined the oxygen dip rate and the MUA synchronization rate as the average number of occurrences of consumption spikes and increases in the MUA above the threshold, over a 30 s (300-time point) time window, with an overlapping window of 299 time points.

### Statistical analysis

To analyze the change in baseline after injection, we used a paired *t*-test to compare the average PO_2_ in the 5 min before injection the average PO_2_ during the 5 min after injection. Our next aim was to statistically compare the high frequency component of the PO_2_ power spectra before and after the injection of PTX. We defined the high frequency to be between 5 and 15 cycles per minute (cpm). Note that this high frequency range, which is nearly always present in the normal adult resting state, is distinguished from the high frequency consumption dips post PTX injections, which occur at a much higher rate of 10 Hz. Using our maximum value scaled power spectra for PO_2_, we calculated the percentage of power over all frequencies within the high frequency range for our specific time windows. This gave us the metric of the proportion of high frequency power for the time windows of 0–10 min and 30–40 min. We then recorded these proportions for those two time windows over all our experiments (PTX and Saline, separately), and used a paired *t*-test to determine if the mean differences in the proportion of power in the high frequency range before and after injection were statistically significant. We also compared the relative amplitude of low frequencies in PO_2_, characterized as the frequencies between 1 and 2 cpm, for the first 10-min time window and the 30–40-min time window. We measured the total power within the 1–2 cpm frequency ranges, scaled relative to the total power for the time window, and applied a paired *t*-test to measure the statistical significance of the mean differences between the 0 to 10-min time window and the 30–40-min time window.

To determine the statistical significance of our oxygen dip rate and MUA synchronization measures for our PTX experiments, we calculated *p*-value thresholds in accordance to the null distribution generated by our saline data. The null distribution for the oxygen dip rate was generated by storing all the oxygen dip rate values for the saline data, across all time windows, and across all saline experiments. The null distribution for the MUA synchronization measure was calculated by the same means. We were then able to construct histograms, and use a best-fit beta distribution to approximate the theoretical distribution of our null data for each metric. Using, for example, the theoretical null distribution of the oxygen dip rate, we were able to determine the value of the oxygen dip rate which corresponded to a *p*-value of 1%. Using the null distribution, we were then able to determine the *p*-value thresholds for our oxygen dip rate and MUA synchronization metrics.

Finally, we analyzed the overshoot after the consumption dips by getting the average across all PTX experiments and using a paired t-test between points before and after the dip. Then the point after the dip was subtracted from the point before the dip to get the average difference which represents the overshoot.

## Results

### Hypoxic dips

**FIGURE 1:**
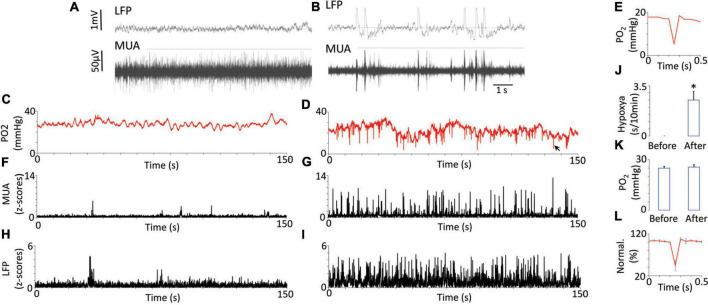
Oxygen dynamics in response to neuronal synchronization induced by the injection of picrotoxin. Examples of raw recording of multi-unit activity (MUA) and local field potentials (LFP) before **(A)** and after **(B)** injection of picrotoxin illustrate electrophysiological characteristics of neuronal synchronization (appearance of rhythmic bursts and near-silent periods between them). Fluctuations of brain tissue oxygen (PO_2_) before injections were relatively periodical with low magnitude **(C)**. After the injection the magnitude of PO_2_ fluctuations increased and regular dips below 10 mmHg appeared **(D)**. Sometimes the dips reached even 5 mmHg while having duration less then 100 ms **(E)**. The black arrow indicates the dip on D which is presented on panel **(E)**. MUA before injection did not have any regular bursts **(F)** but after injection the bursts appeared **(G)**. LFP before **(H)** and after injection **(I)** generally followed the behavior of MUA. Note that dips in PO_2_ appeared together with bursts on MUA or LFP. The total duration of hypoxia reached 2 s over 10 min **(J)**. The averaged baseline of PO_2_ did not change after the injection (*N* = 7) **(K)**. The error bars represent standard error. The overshoot after the dip is shown on the population data **(L)**. The data was normalized to 100% of the point before the dip. Error bars represent standard deviation for better visibility. Asterisk represents a significant difference (*p* < 0.05).

Neuronal hypersynchronization events (Figures 1A,B) and multiple dips in brain tissue PO_2_ were recorded after picrotoxin injections (Figures 1C,D). The occurrences of these dips corresponded to the synchronized bursts in neuronal activity on LFP/MUA (Figures 1F–I), and they were never observed before picrotoxin injection or after an injection of a control vehicle. We did not observe any head motion associated with synchronized bursts of neuronal activity or any seizures. The synchronized LFP bursts had a duration of around 80 ms whereas the dips in PO_2_ had durations of approximately 30–40 ms (Figure 1E and Supplementary Figure 1). Based on (Ougorets and Caronna, 2008), we took the threshold critical/hypoxic level to be 10 mmHg. The cumulative duration of dips over 10 min was 2.5 ± 0.72 s (Figure 1J). None of these events were observed before the injection of picrotoxin or after the injection of vehicle and, thus, a two-tailed paired t-test showed significance (*p* < 0.013). The baseline of brain tissue oxygen, measured as the time-average, did not change after picrotoxin injection (24.89 ± 1.1 mmHg before vs. 25.52 ± 1.49 mmHg after, mean ± SEM) (Figure 1K). Supplementary Figure 2 additionally illustrates the relationship between raw data of PO_2_ and electrophysiology after injection of picrotoxin.

An overshoot was often observed after the dips (Figure 1E). A two-tailed paired t-test comparing points before and after the dip revealed a significant difference (*p* = 0.007). On average the difference between levels of oxygen which preceded and followed the dip was 1.28 ± 0.21 mmHg (mean ± SEM). Sometimes this difference could reach 4 mmHg. Figure 1L shows group data normalized to the value before the dip.

### Power spectra analysis for PO_2_ and local field potentials

**FIGURE 2:**
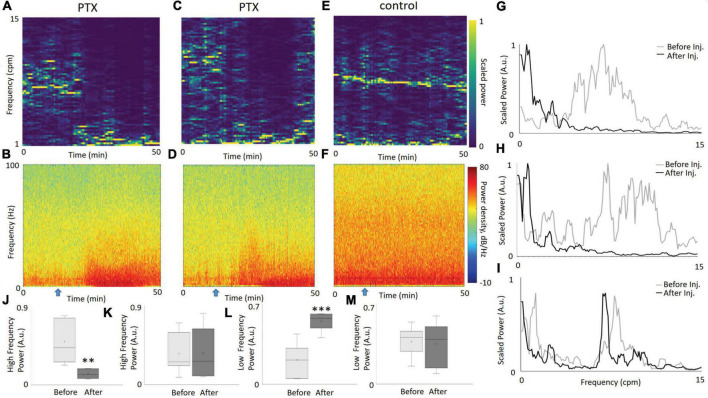
The dynamics of PO_2_ and LFP, before and after the injection of PTX. PO_2_ spectrograms from two illustrative experiments **(A,C)** highlight the change of dominant frequencies from relatively fast (5–15 cpm) to slow (<5cpm) after injection of PTX at the 15 min mark (denoted by a blue arrow). An increase in the power of the delta LFP band, as seen in the LFP spectrograms **(B,D)**, following the injection. Meanwhile, both the PO_2_ and LFP spectrogram for the saline experiment show no apparent change in dynamics [**(E,F)**, respectively]. Comparing the power spectral density (PSD) of PO_2_ from the first 10 min to a time window between 30 and 40 min, explicitly shows the disappearance of high frequency PO_2_ fluctuations for the PTX experiments [**(G,H)**, corresponding to **(A,C)**, respectively], unlike the saline experiment corresponding to panel **(E)**, which displays no visible change **(I)**. We measured the proportion of power within the PSD in the 5–15 cpm frequency (“high frequency”) range for periods before and after injection, over all our PTX experiments (*N* = 7) and display the distribution as a whisker-bar plot **(J)**. A paired *t*-test confirmed that the difference in proportion of the high frequency component before and after injection is statistically significant (*p* = 0.002). Similarly, for our saline experiments (*N* = 7), there was no statistical difference before and after injection **(K)**, confirming the stability of a high frequency PO_2_ oscillatory component in the control. We also compared the low frequency (1–2 cpm) amplitude relative to the total power for each time window, graphing a bar-whisker plot for our PTX data **(L)** and our saline data **(M)**. The differences in amplitude are statistically significant (*p* = 0.0008) before and after injection for our PTX experiments, and not significant for our saline experiments. Two asterisks represent a significant difference *p* < 0.01. Three asterisks represent a significant difference *p* < 0.001.

Looking over the entire duration of the experiment, the injection of PTX had a distinct effect on the overall frequency distribution of PO_2_. As the PO_2_ spectrograms show (Figures 2A,C), the power at higher frequencies greatly decreased during a certain period after injection (occurring at 15 min). As expected, this decrease in higher frequency fluctuations corresponds with an increase in the delta LFP band power (Figures 2B,D), signifying neuronal hypersynchronization induced by PTX. In contrast, for our control experiment, there is no visible change in the dynamics of the high frequency component of our PO_2_ time series (Figure 2E) nor in the power of any particular LFP band (Figure 2F). The rapid increase in the delta band power, with a simultaneous decrease in the high frequency power of PO_2_, in contrast to the control case, highlights the link between the onset of neuronal hypersynchronization and a disruption in brain tissue oxygen fluctuations via a shift in PO_2_ power spectra toward the lower frequencies.

Figures 2G,H show the power spectra diagrams for two separate PTX experiments. Figure 2I shows the power spectra for the same two time windows for the control experiment. The power shifts from high frequencies to lower frequencies for the PTX experiments, and stays the same for the control experiment. To visualize this phenomenon over all PTX experiments, we calculated the proportion of power contained within the 5-15 cpm frequency range (we denote this as high frequency) for both before and after injection, and graphed the box-whisker plot of their distribution (Figure 2J). We applied the same methodology over our saline experiments, and graphed the resulting box-whisker plots (Figure 2K). A paired *t*-test confirms that the mean difference of the proportion of high frequency power before and after PTX injection is statistically significant (*p* = 0.0028), while the control data shows no statistically significant difference before and after saline injection (*p* = 0.94). Similarly, we calculated the proportion of power within the 1–2 cpm frequency range (denoted as low frequency) for the respective before and after injection time windows for both our PTX experiments (Figure 2L) and saline experiments (Figure 2M). The mean differences in slow frequency amplitudes before and after injection for our PTX experiments was statistically significant (p = 0.0008), while the saline experiments showed no statistical difference. Supplementary Figure 3 illustrates the change in total (not relative) power and indicates that the amplitude of slow frequencies (1–2 cpm) greatly increased after injection of picrotoxin. Supplementary Figure 4 illustrates similar changes in power when a 200Hz sampling frequency was used.

### Consumption and synchronization dynamics

**FIGURE 3:**
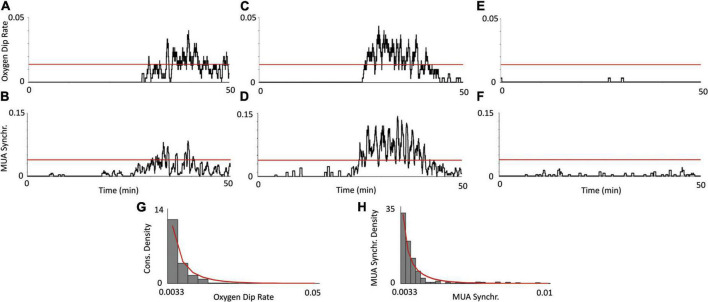
Relationship between oxygen consumption and neuronal synchronization. Using the same two PTX experiments from Figure 2, a plot of the consumption rate measure **(A,C)**, shows a significant increase in PO_2_ consumption past the 30 s mark. This increase in PO_2_ consumption corresponds with an increase in MUA activity **(B,D)**, indicating that the consumption spikes are indeed due to an increase in aggregate neuronal activity. The oxygen dip rate and MUA synchronization measure show no statistically significant deviations [**(E,F)**, respectively], indicating that this consumption activity does not occur in the control. The red, horizontal line denotes the threshold value (for each respective measure) at a 1% level of significance, calculated from the distribution of saline experiments. The density plot of the oxygen dip rate and MUA synchronization measures over all saline experiments (*N* = 7) is plotted [**(G,H)**, respectively]. The bars denote data from our actual recorded control experiments and the red line denotes the best-fit beta distribution.

Using the same two PTX experiments and control experiment as in Figure 2, we plot the dynamics of the oxygen dip rate alongside with MUA synchronization (Figures 3A–F). The oxygen dip rate is a measure of the average number of consumption spikes over a rolling 30 s window. For our PTX experiments (Figures 3A,C), there is a sudden increase in the oxygen dip rate after 30 min with multiple values crossing the 1% *p*-value threshold (red line). The control experiment has a oxygen dip rate of nearly zero over the entire 50-min duration (Figure 3E). MUA synchronization measures the average number of significant increases in the absolute value of the original MUA signal. For our PTX experiments, a significant increase in MUA synchronization corresponds to increases in the oxygen dip rate (Figures 3B,D), while the control experiment shows no significant increase in the level of MUA synchronization (Figure 3F). Figures 3G,H show the density plots for the oxygen dip rate and MUA synchronization measures over all saline experiments, respectively. The red line plots the best-fit beta distribution over the recorded data, representing the theoretical distribution used to calculate the *p*-value threshold. The calibration PO_2_ values are shown by Supplementary Figure 5.

## Discussion

Our results show that pharmacologically mimicking the debilitation of interneurons produced two phenomena at the level of brain tissue oxygen: (1) there was a change in the rate and magnitude of brain tissue oxygen fluctuations and (2) there were short-lasting and severe dips in PO_2_. The interaction between these events caused localized hypoxia.

The fluctuation of brain tissue PO_2_ in the brain is an important and ever-present process that corresponds to the vasomotion at the level of arterioles (Hudetz et al., 1998; Aalkjaer et al., 2011). These fluctuations are thought to prevent local hypoxia (Tsai and Intaglietta, 1993; Goldman and Popel, 2001; Doubovikov and Aksenov, 2020). In the normal brain, the predominant frequency is around 10 cpm in adults (Manil et al., 1984; Linsenmeier et al., 2016; Aksenov et al., 2018), and this frequency is an indicator of mature neurovascular interactions which allow fast responses to localized increases in neuronal activity (Doubovikov and Aksenov, 2020). Injections of picrotoxin shifted the dominant frequency of oxygen fluctuations toward 1–3 cpm by increasing their magnitude. Interestingly, this slow frequency corresponds to the “neonatal” frequency of oxygen fluctuations where neuronal control over vasomotion is yet to be established and arteriolar vasomotion is spontaneous (Aksenov et al., 2018). Moreover, this slow frequency dominates under general anesthesia (Linsenmeier et al., 2016), when neuronal activity is greatly decreased (Aksenov et al., 2015). Thus, it indicates that the full function of local neuronal networks is required for oxygen fluctuations of higher frequencies (∼10 cpm) to dominate. Since picrotoxin causes a “disconnection” not only between interneurons and pyramidal cells but also between interneurons and arterioles (Vaucher et al., 2000), these processes disturb neuronal control of vasomotion, which leads to the observed shift of brain tissue oxygen fluctuations toward lower frequencies.

The other observed phenomenon, short-duration (<100 ms) dips in brain tissue oxygen, is most likely related to elevated energy consumption when neuronal activity is synchronized. An oxygen decrease was also observed during stimulation, which was accompanied by a delayed vascular response (Uludag, 2008; Watanabe et al., 2013; Freeman and Li, 2016). It is necessary to emphasize that epileptiform neuronal bursts are different from neuronal responses to stimulation: the bursts represent a hypersynchronization of the total neuronal population; they consist of sustained neuronal depolarization with multiple action potentials following each other (Bromfield et al., 2006) and, thus, indicate very fast, brief and massive oxygen consumption, which can be observed even *in vitro* (Ivanov et al., 2015), depending how fast the oxygen electrode can respond. Our results clearly indicated there to be periods of localized hypoxia where the oxygen level was below 10 mmHg, and at times they even dropped to 5 mmHg, indicative of a more severe hypoxic event. Note that there is not a generally accepted level designated as hypoxia, and the values can be different depending on experimental conditions (Kariman et al., 1983; Foster et al., 2005; Kasischke et al., 2011). Overall, our results point to the role of interneurons and GABA in regulating neuronal activity by preventing resting-state neuronal hypersynchronization and the corresponding hypoxia in brain tissue.

It is generally accepted that the vascular response is typically delayed by 2–5 s (Zheng et al., 2002; Masamoto et al., 2003; Aksenov et al., 2015; Cai et al., 2018), which raises the question of what mechanism is responsible for recovery from the short-lasting resting state dips in brain tissue oxygen. Only three processes can possibly explain the recovery: vascular delivery of oxygen, oxygen diffusion from neighboring tissue and a drop in oxygen consumption while maintaining constant oxygen delivery. The role of oxygen diffusion from neighboring tissue can be excluded due to its very slow rate, dependent on an O_2_ diffusion coefficient of 1540 μm^2^/s in the brain (Ganfield et al., 1970). Calculations show that the actual oxygen diffusion rate is 2 μm/50 ms in our case, which is not remotely sufficient to compensate for oxygen loss, taking into account the distances between our tubing/electrodes (150–200 μm). On the other hand, we have observed a small overshoot in dips (Figure 1), which directly indicates the role of the vascular response, a drop in the oxygen consumption or some combination of both. Such a quick vascular response is interesting and is possible due to the physiology of the smooth muscle cells of arterioles, because they can relax in less than 50 ms (Nelson et al., 1995; Hall, 2011) and involve fast-acting mediators (Hosford and Gourine, 2019). However, the drop in oxygen consumption is also possibly due to neurons entering a hyperpolarized phase, following the epileptiform burst. Additional studies would be necessary to establish the exact timing difference between LFP spikes and PO_2_ dips by recording at higher frequency and to ascertain whether this is a region-dependent effect.

The short duration of hypoxia points to the question of whether it can produce actual damage. Generally speaking, with sufficient severity, hypoxia can lead to the upregulation of reactive oxygen species (ROS) which can rapidly accumulate beyond the protective capacity of the antioxidative system, leading to oxidative stress (Chen et al., 2018). The presence of oxidative stress poses a potential detrimental threat to a range of cell types in the brain, as the excess of ROS has a high propensity to react with and damage macromolecules within cells (e.g., DNA/RNA oxidation, protein oxidation, nitration of tyrosine residues, and lipid peroxidation), leading to the debilitation of the cell (Moreira et al., 2005). Moreover, hypoxic conditions have also been shown to lower intracellular and extracellular pH(Rolett et al., 2000; Yao and Haddad, 2004), phosphocreatine (Rolett et al., 2000), inorganic phosphate (Nioka et al., 1990; Rolett et al., 2000), lactate (Sarrafzadeh et al., 2003), as well as NADH (Rolett et al., 2000; Shetty et al., 2014). The combination of these consequences to the biochemical environment of affected cells can significantly impair their normal functioning and are associated with subsequent neuroapoptosis. However, it is difficult to suggest such dramatic changes to be present during the short duration of hypoxia we observed in this study, because the results of previous studies are predominantly based on measurements taken minutes after the onset of ischemia. On the other hand, processes related to oxidative stress are very fast and can take place within milliseconds. For example, it has been shown that a significant burst of local ROS occurs within 2 s of a mitochondrial permeability transition event, which, in turn, could have immediate consequences for the local cellular homeostasis (Zorov et al., 2000). Although it is difficult to compare the relative effects of these smaller, harmful events to hypoxia under longer durations, the repetitive nature of the short-lasting hypoxia due to the debilitation of interneurons can potentially have an accumulating effect. Our data suggest that over the course of 10 min, there will be an accumulated 2 s of hypoxic conditions (Figure 1). Moreover, considering this effect can persist for years in humans, the number of such short hypoxic events can be tremendous.

Finally, we would like to emphasize that due to the short duration of the reported hypoxic events, not all methods can be used to record and observe them. It seems that the task of recording brain tissue oxygen requires a minimum of a 20 Hz sampling rate. This excludes methods, such as functional MRI, that do not have sufficient temporal resolution. However, subclinical local neuronal hypersynchronization can be visible on EEG directly (depending on the size of epileptogenic zone) and indirectly (where slow bands (e.g., delta) prevail locally during the awake resting state).

We conclude that excitatory-inhibitory imbalance and long-lasting resting state neuronal hypersynchronization is a potentially dangerous phenomenon because of the multiple events of local hypoxia. In summary, we have shown the importance of interneurons in maintaining both physiological levels of neuronal activity and oxygen homeostasis. Interneurons are involved in regulating oxygen delivery through brain tissue oxygen fluctuations and oxygen consumption via restricting synchronized neuronal activity in resting state.

## Data availability statement

The raw data supporting the conclusions of this article will be made available by the authors, without undue reservation.

## Ethics statement

The animal study was reviewed and approved by NorthShore University HealthSystem Research Institute Institutional Animal Care and Use Committee.

## Author contributions

DA: conceptualization, project administration, and resources. ED, DA, and NS: formal analysis. DA and AD: funding acquisition and supervision. NS and DA: data acquisition. DA and RL: methodology. ED: software. DA, ED, and DG: roles/writing—original draft. NS, RL, and AD: writing—review and editing. All authors contributed to the article and approved the submitted version.
