# Supplementary material for: Brain tissue oxygen dynamics while mimicking the functional deficiency of interneurons
Source: Front Cell Neurosci. 2022 Oct 20;16:983298. doi: 10.3389/fncel.2022.983298 (PMC9630360; doi:10.3389/fncel.2022.983298)
Supplement: Supplementary file 1 [file Data_Sheet_1.pdf]

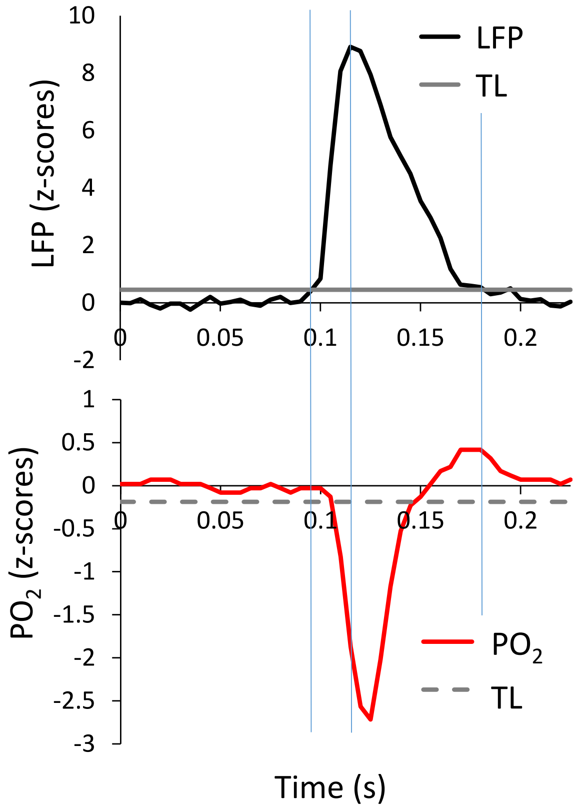

Figure S1. The timing between an LFP burst and PO_2 dip after the injection of picrotoxin. Grey lines (solid and dashed) represent tolerance limits (TL) indicating that with 95% confidence, 99% of points will be below (LFP) or above (PO_2) TL lines. TL is used to define the onset and offset of the LFP burst and PO_2 dip. The PO_2 data were acquired at a 200Hz sampling rate, and the LFP data was digitized at 200Hz. The blue lines were placed at the intersection between the onset, peak and the end of the positive LFP burst. The distance between the peaks of LFP and PO_2 is 10-20 ms.

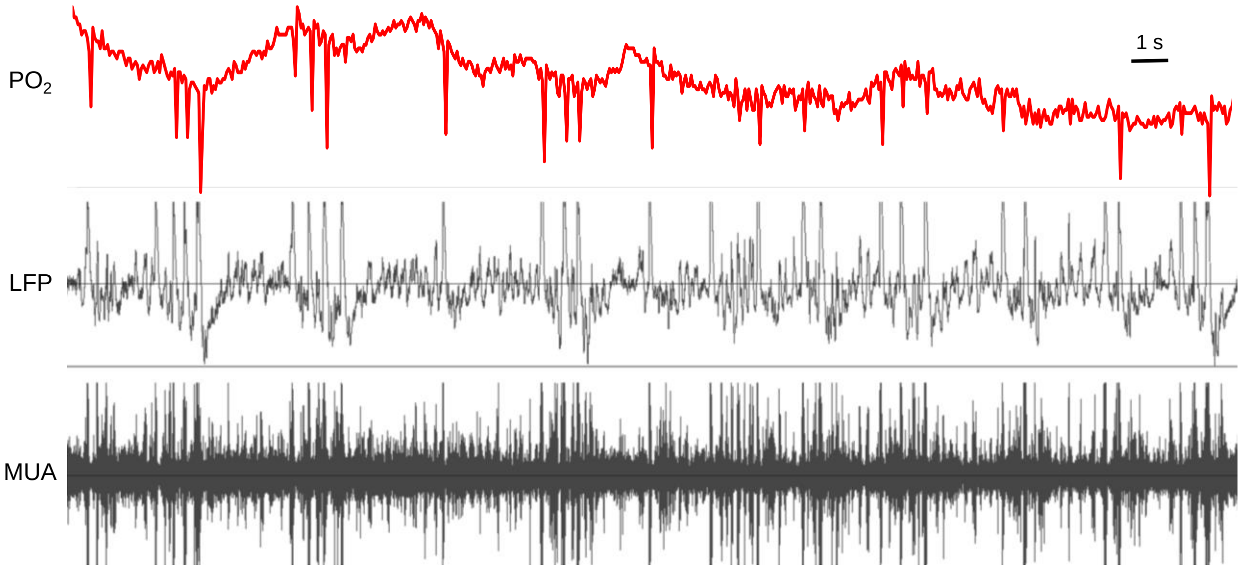

Figure S2. An example of raw simultaneous recording of PO_2 and electrophysiology after injection of picrotoxin. The dips in PO_2 visibly correspond to synchronization bursts in neuronal activity.

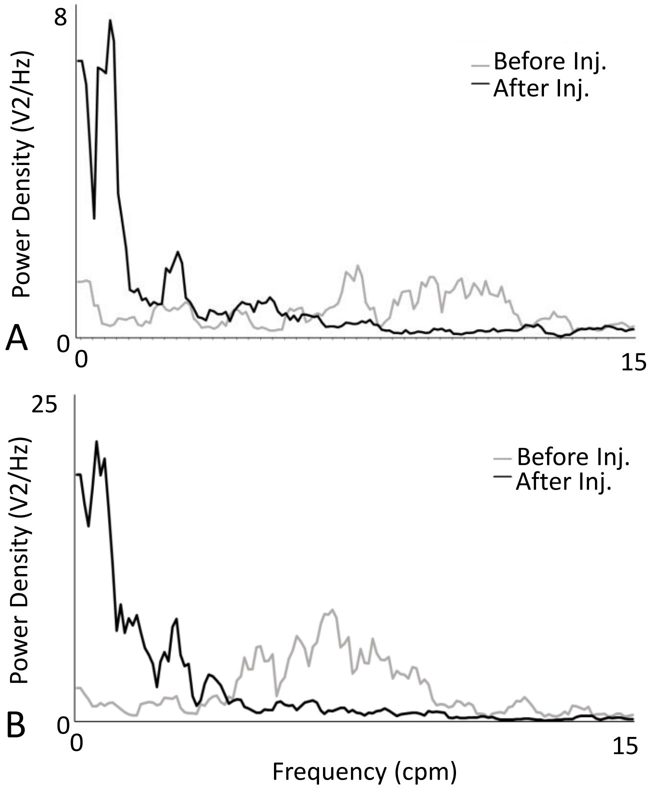

Figure S3. Change in power density before and after injection of picrotoxin. Using the same two picrotoxin experiments as in Figure 2, we plot the power spectra in the 0-10 minute time window (before injection) and the 30-40 minute time window (after injection). The grey and black lines denote before injection and after injection, respectively. We use power density (power/frequency) over a frequency range of 0-15 cpm. Panel A corresponds to Fig 2G and panel B corresponds to Fig 2H. In both experiments there is a significant increase in the absolute power at the low frequencies (1-2 cpm) after injection.

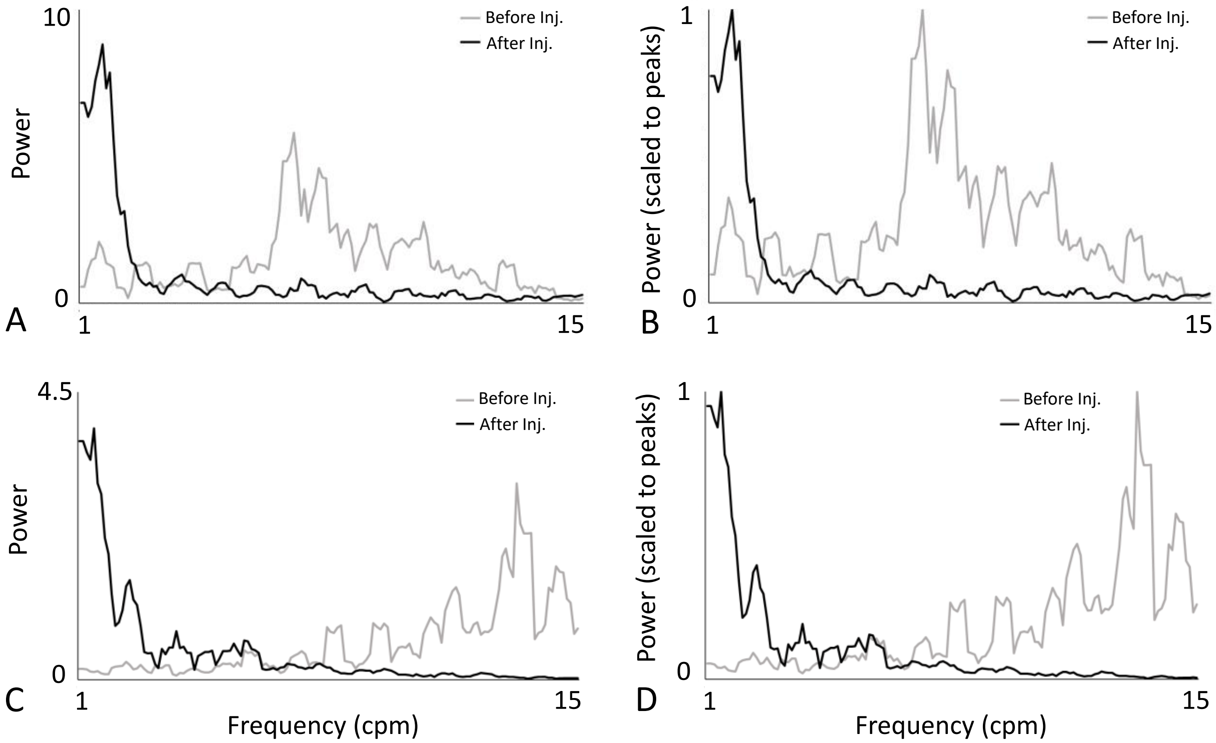

Figure S4. Change in power density before and after injection of picrotoxin. Using the additional two picrotoxin experiments (400Hz low-pass filter of the analog signal and 200Hz sampling rate), we plot the power spectra in the 0-10 minute time window (before injection) and the 30-40 minute time window (after injection). The grey and black lines denote before injection and after injection, respectively. We use power density (power/frequency) over a frequency range of 0-15 cpm. In both experiments there is an increase in the absolute (A, C) and relative (scaled to peaks) (B, D) power for the low frequencies (1-2 cpm) after injection.

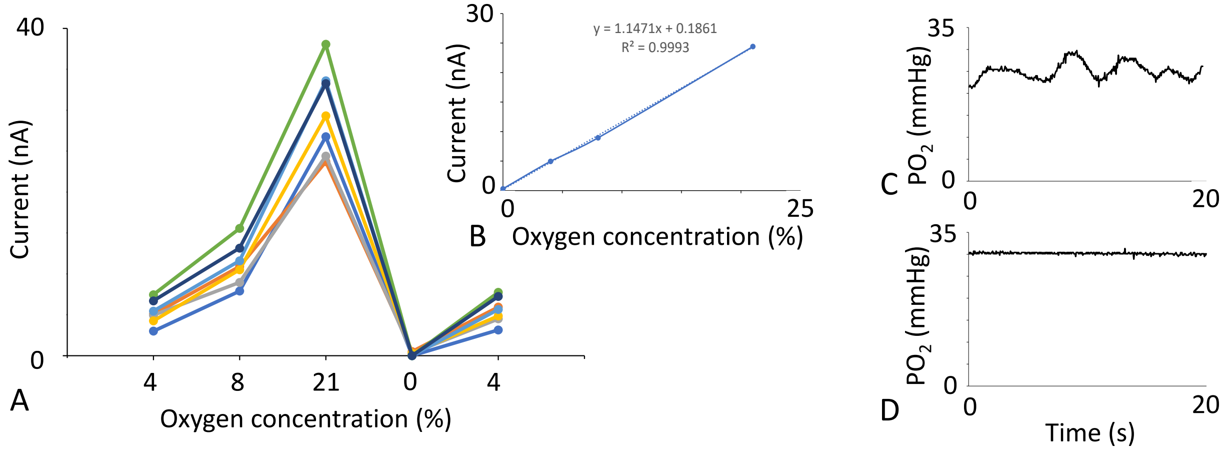

Fig. S5. Electrode calibration. Basic calibration values of the oxygen electrodes (N=7), when operated as static concentration sensors (A). Calibration solutions were made using saline bubbled with fixed gas mixtures. The calibration procedure was performed in a closed chamber and consisted of 1.5 min of bubbling with different concentrations of oxygen (4%, 8%, 21%, and 4% for a second time, which was used as a control). The 0% oxygen concentration was achieved by bubbling with 100% N_2 for 2.5 min. The order of the calibration gases is shown (A). After the bubbling stopped, the PO_2 was measured for 20s before switching to a different oxygen concentration. An example of a calibration curve is shown (B). Two examples of PO_2 recordings (C – in awake rabbit, D – in calibration chamber using 30mmHg of oxygen) are shown for the same electrode. Note that oxygen oscillations are absent when the recording was conducted in the calibration chamber.
